# Inferring time derivatives including cell growth rates using Gaussian processes

**DOI:** 10.1038/ncomms13766

**Published:** 2016-12-12

**Authors:** Peter S. Swain, Keiran Stevenson, Allen Leary, Luis F. Montano-Gutierrez, Ivan B.N. Clark, Jackie Vogel, Teuta Pilizota

**Affiliations:** 1 SynthSys—Synthetic and Systems Biology, School of Biological Sciences, University of Edinburgh, Mayfield Road, Edinburgh EH9 3BF, UK; 2 Department of Biology, McGill University, Montreal, Quebec, Canada H3G 0B1; 3 Integrated Quantitative Biology Initiative, McGill University, Montreal, Quebec, Canada H3G 0B1

## Abstract

Often the time derivative of a measured variable is of as much interest as the variable itself. For a growing population of biological cells, for example, the population's growth rate is typically more important than its size. Here we introduce a non-parametric method to infer first and second time derivatives as a function of time from time-series data. Our approach is based on Gaussian processes and applies to a wide range of data. In tests, the method is at least as accurate as others, but has several advantages: it estimates errors both in the inference and in any summary statistics, such as lag times, and allows interpolation with the corresponding error estimation. As illustrations, we infer growth rates of microbial cells, the rate of assembly of an amyloid fibril and both the speed and acceleration of two separating spindle pole bodies. Our algorithm should thus be broadly applicable.

## Abstract (web summary)

High-throughput time-series data is increasingly available, yet estimating time-derivatives from such data can remain a challenge. Here, the authors provide a non-parametric method for inferring the first and second time-derivatives from multiple replicates of time-series data and for estimating errors in this inference and in any summary statistics.

Estimating the time derivatives of a signal is a common task in science. A well-known example is the growth rate of a population of cells, which is defined as the time derivative of the logarithm of the population size^1^ and is used extensively in both the life sciences and biotechnology.

A common approach to estimate such derivatives is to fit a mathematical equation that, say, describes cellular growth and so determine the maximum growth rate from the best-fit value of a parameter in the equation^2^. Such parametric approaches rely, however, on the mathematical model being a suitable description of the underlying biological or physical process and, at least for cellular growth, it is common to find examples where the standard models are not appropriate^3^.

The alternative is to use a non-parameteric method and thus estimate time derivatives directly from the data. Examples include taking numerical derivatives^4^ or using local polynomial or spline estimators^5^. Although these approaches do not require knowledge of the underlying process, it can be difficult to determine the error in their estimation^5^ and to incorporate experimental replicates, which with wide access to high-throughput technologies, are now the norm.

Here we develop a methodology that uses Gaussian processes to infer both the first and second time derivatives from time-series data. One advantage of using Gaussian processes over parametric approaches is that we can fit a wider variety of data. Rather than assuming that a particular function characterizes the data (a particular mathematical equation), we instead make assumptions about the family of functions that can describe the data. An infinite number of functions exist in this family and the family can capture many more temporal trends in the data than any one equation. The advantages over existing non-parametric methods are that we can straightforwardly and systematically combine data from replicate experiments (by simply pooling all data sets) and predict errors both in the estimations of derivatives and in any summary statistics. A potential disadvantage because we use Gaussian processes is that we must assume that the measurement noise has a normal or log-normal distribution (as do many other methods), but we can relax this assumption if there are multiple experimental replicates.

To illustrate how our approach predicts errors and can combine information from experimental replicates, we first focus on inferring growth rate from measurements of the optical density of a growing population of biological cells. Plate readers, which are now widespread, make such data easy to obtain, typically with hundreds of measurements and often at least three to ten replicates. We will also, though, show other examples: estimating the rate of *in vitro* assembly of an amyloid fibril and inferring the speed and acceleration of two separating spindle pole bodies in a single yeast cell.

## Results

### An overview of Gaussian processes

A Gaussian process is a collection of random variables for which any subset has a joint Gaussian distribution^6^. This joint distribution is characterized by its mean and its covariance.

**Figure 1:**
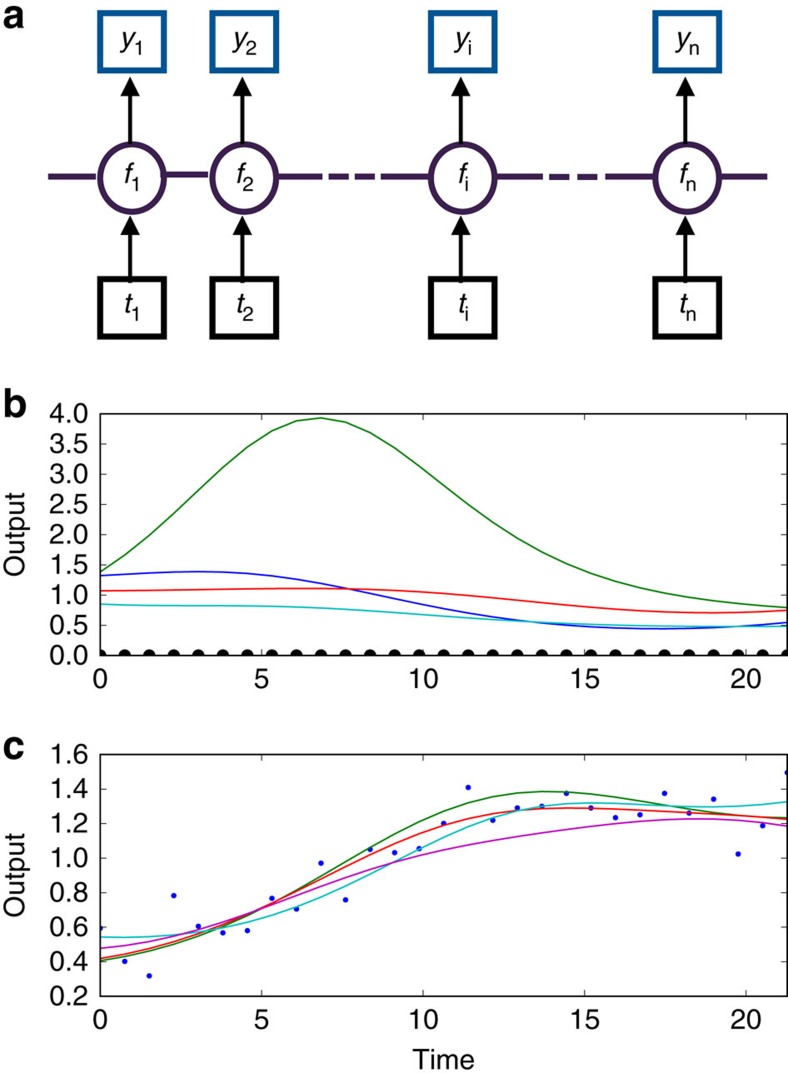
An overview of inference with Gaussian processes. (**a**) A graphical model of a Gaussian process^6^. Squares denote known variables (times, *t*_*i*_, and data points, *y*_*i*_); circles denote unknown variables (the underlying, latent function, *f*_*i*_). We associate a variable in the Gaussian process to each time point and the value of this variable gives the value of the latent function. Each observed data point, *y*_*i*_, depends only on the corresponding latent variable, *f*_*i*_. Each *f* variable, however, depends on all the other *f* variables (they covary). (**b**) Four examples of latent functions with a squared exponential covariance function. The functions are strictly only defined at the time points of the observations (shown with black semi-circles on the *x* axis) but are drawn with a continuous line for clarity. (**c**) Four examples of latent functions after conditioning on the data (data are shown as blue dots). Although each individual function is smooth, there is more variation between functions where the data is more spread. Averaging many latent functions gives the best fit. The hyperparameters of the covariance matrix are the same as those in **b**.

To use a Gaussian process for inference on time series, we assume that the data can be described by an underlying, or latent, function and we wish to infer this latent function given the observed data. For each time point of interest, we add a random variable to the Gaussian process. With *n* time points, there are therefore *n* corresponding random variables in the Gaussian process. The latent function is given by the values taken by these random variables (Fig. 1a). Without losing any generality, we set the mean of each random variable to be zero^6^.

How each random variable in the Gaussian process depends on the other random variables both at earlier and later times, that is, how the variables covary, determines the shape of the latent function. For example, if each random variable does not covary with any other (the covariance matrix of the Gaussian process is the identity matrix), then the latent function will randomly jump back and forth around zero. If each random variable covaries equally with every other random variable (all the entries of the covariance matrix are one), then the functions sampled will be straight horizontal lines starting at the value of the random variable associated with the first time point. More pertinently, if the covariance for each random variable is positive for those random variables close in time and tending to zero for random variables far away in time, then the functions generated vary, but do so smoothly.

To proceed, we therefore must choose the type of covariance function for the Gaussian process and, in doing so, we necessarily make some assumptions about the latent functions that underlie the data. We will often use a squared exponential covariance, which imposes little restriction on the shape of the latent function other than to assume that it is smooth (infinitely differentiable; Fig. 1b). The Matern covariance function (paramaterized with *ν*=5/2) relaxes this smoothness assumption and imposes that the latent function is only twice differentiable^6^. Another alternative is the neural network covariance, which tends to generate sigmoid-shaped latent functions^6^. We emphasize that choosing a covariance function to describe the latent function puts much less restriction on its shape than the more traditional choice of selecting a particular mathematical equation to model the latent function.

Each covariance function is parameterized in its own way and we must find the appropriate values of these parameters given the data. More correctly, the parameters are called hyperparameters (Methods) and determining the hyperparameters is the computationally intensive part of the inference.

Once the hyperparameters are optimized, we can sample from the Gaussian process to generate a latent function that is consistent with our data (Fig. 1c). The mean latent function, which we could find from averaging many samples but can also calculate directly, gives the ‘best-fit' and the variance of the latent function provides an estimation of the error in the inference.

Following on from earlier work^7^, we adapt this standard inference procedure to also allow the inference of the first two time derivatives of the latent function, because these time derivatives are, in many applications, of more interest than the latent function itself. Errors in inference of the latent function are automatically carried through to the errors in inferring time derivatives.

### Verification of the algorithm

**Figure 2:**
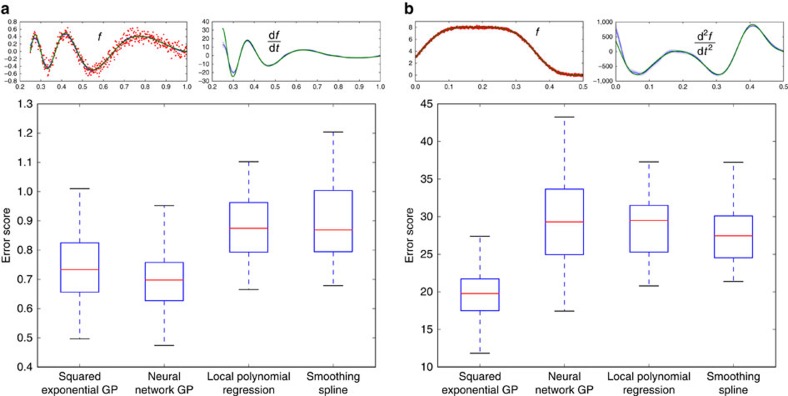
The inference method can perform better than alternatives. (**a**) Inference of the first derivative. A box plot of error scores (related to the mean absolute difference between the inferred and exact derivative) for inference of the first derivative. We use either a squared exponential covariance function or a neural network covariance function for the Gaussian process (GP) and compare with local polynomial regression (with *p*=3) and a quintic penalized smoothing spline (data for both from ref. 8). Top left shows one sample data set (in red with 500 data points), the true underlying function (in green) and the inferred latent function using a neural network covariance function—the best fit (in blue); top right shows the corresponding first derivative (with here an error score of 0.64): exact (in green) and inferred (in blue). Equivalent plots for the alternative inference methods are given by De Brabanter *et al*.^8^. Errors (in light blue) are s.d. (**b**) Inference of the second derivative. A box plot of scores for inference of the second derivative. The two alternatives are local polynomial regression (with *p*=5) and a septic penalized smoothing spline (data for both from ref. 8). Top right shows one sample data set (in red with 1,500 data points), the underlying function (in green) and the inferred latent function using a neural network covariance function (in blue); top left shows the corresponding second derivative (with here an error score of 26.2): exact (in green) and inferred (in blue).

To verify our algorithm's inference of first and second time derivatives, we followed the tests of De Brabanter *et al*.^8^. Gaussian measurement noise was added to the same analytic functions chosen by De Brabanter *et al*.^8^ for which time derivatives can be found exactly and the mean absolute difference between the inferred derivative and the exact derivative was used to score the inference (see ref. 8 for details—the end points are not included). We show the distribution of scores for 100 different data sets each with a different sample of the measurement noise (Fig. 2).

For these tests, our method outperforms established alternatives. For illustration, we show results for both the squared exponential covariance function and the neural network covariance function. Independent of the choice, the method performs at least as well as alternatives (Fig. 2).

### Estimating cellular growth rates

We now consider the inference of microbial growth rates (strictly, we infer the specific growth rate: the time derivative of the logarithm of the population size). The population size as a function of time is commonly fit to a parametric equation^2^, although these equations are restrictive and describe only a particular type of growth^3^. Therefore, to provide a further test of our algorithm, we considered a linear sum of two growth equations—the Gompertz^9^ and Richards^10^ models—to generate a growth curve that cannot in principle be fit by either, but where an exact expression for the first derivative can still be found. We compare our results with smoothing splines, an established non-parametric alternative^3^.

**Figure 3:**
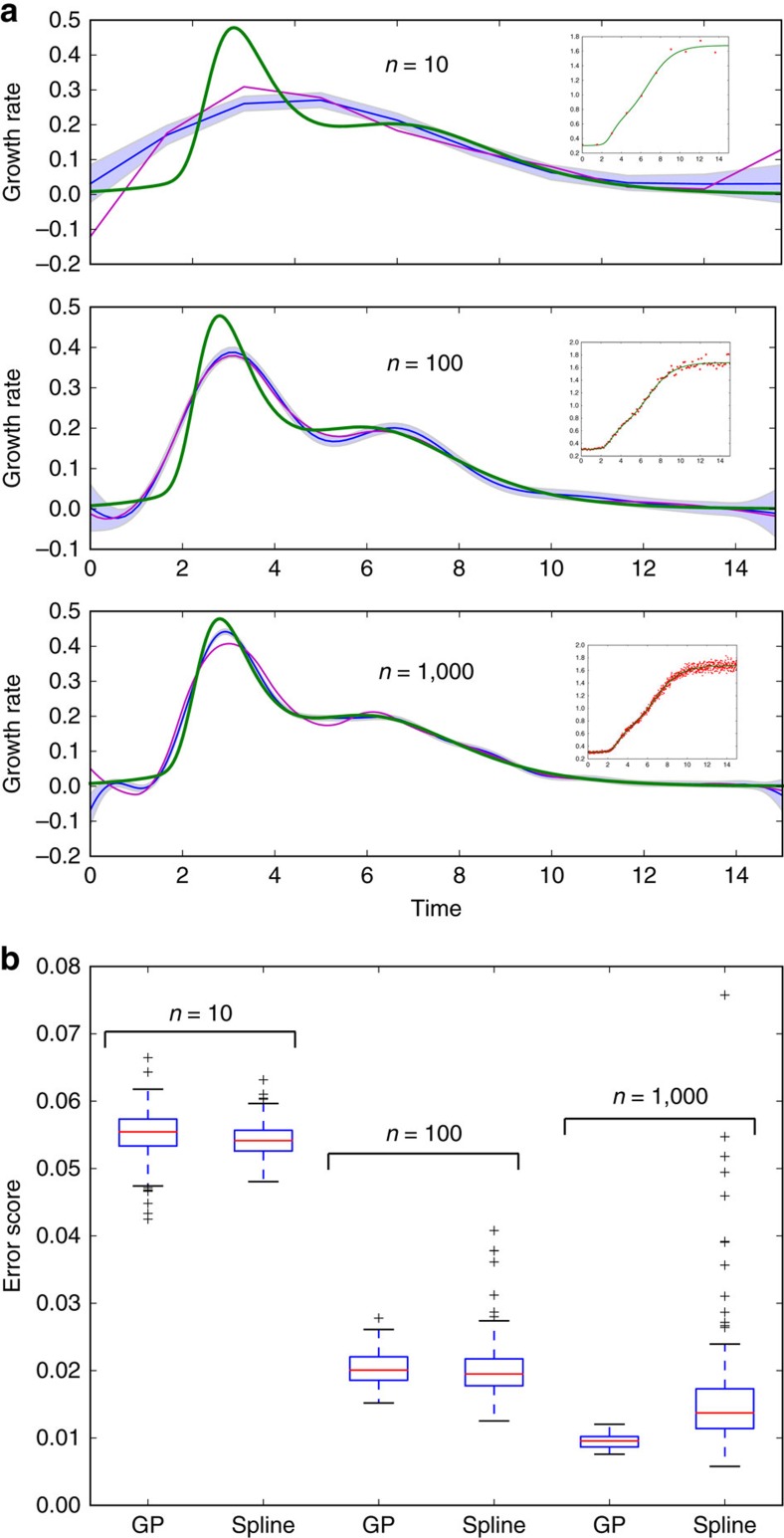
Inference of growth rates with Gaussian processes becomes more robust with increasing numbers of data points. (**a**) We show the inferred growth rate (the best fit with a squared exponential Gaussian process in blue with errors in light blue and from a cubic smoothing spline in purple) and the exact growth rate (in green) for synthetic data sets with either 10, 100 or 1,000 time points (insets, with the underlying growth curve in green). Even though we favour smoothing splines by setting the smoothing parameter to be proportional to the exact variance of the measurement noise (whereas the Gaussian process infers this parameter), both methods perform similarly. (**b**) A box plot of errors scores for inference of the growth rate from 100 data sets with randomly generated log-normal measurement noise. For the Gaussian process, the distribution of error scores tightens with increasing number of data points.

For these data sets and this magnitude of measurement noise, both methods perform equally well, but the inference using Gaussian processes becomes more robust as the number of data points increase (Fig. 3). We note that we have artificially favoured the smoothing spline, because the smoothing parameter for the spline is set with the variance used to generate the synthetic measurement noise. The Gaussian process methodology, in contrast, infers this variance. Despite the advantage of the spline-based inference, its median error is ∼45% higher when *n*=1,000.

**Figure 4:**
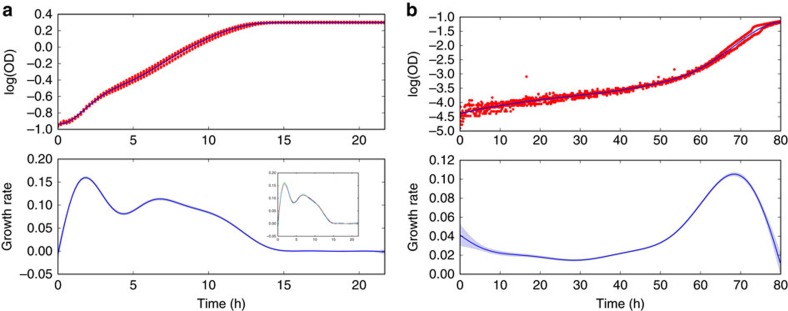
Microbial growth rates can be inferred as a function of time. (**a**) A growth curve of *S. cerevisiae* in a mixture of 0.4% glucose and 1% galactose showing a diauxic shift (7 replicates, each with *n*=115). The best-fit (mean) latent function is shown in dark blue and the inferred growth rate is shown below. All error bars (light blue) are s.d. The inset shows, as an example, four sample estimates of the growth rate as a function of time (samples of the first derivative of the latent function—the corresponding samples of the latent function itself are not shown). (**b**) Growth of *E. coli* in hyperosmotic conditions with an unusually long lag and short growth period (two replicates, each with *n*=646) and the inferred growth rate. The magnitude of the measurement noise is here allowed to vary with time and empirically estimated across the replicates (Methods). Error bars (light blue) are s.d.

Turning to experimental measurements, we fit optical densities, which are proportional to the number of cells if properly calibrated^11^,^12^, and show that we can infer growth rates for two cases that cannot be easily described by parametric approaches^3^. The first exhibits a diauxic shift with two distinct phases of growth and the second shows an exceptionally long lag (Fig. 4). We infer the growth rate and the estimated errors in our inference as a function of time using all experimental replicates. Data from replicate measurements are pooled together and the algorithm applied as for a single replicate.

Having the inferred growth rate over time can make identifying different stages of the growth curve substantially easier than making this identification from the optical density data alone. For example, the local minimum in the growth rate of Fig. 4a is expected to indicate a shift from cells using glucose to using galactose. Inferring a time-dependent growth rate should increase the robustness of high-throughput automated studies, which usually focus on identifying exponential growth^13^,^14^.

Often summary statistics are used to describe a growth curve, such as the maximum growth rate and the lag time^2^, and we can estimate such statistics and their associated errors. From our inference, we can sample latent functions that are consistent with the data. Each sample provides an example of a latent function that ‘fits' the data. To estimate errors in statistics, we generate say 100 samples of the latent function and its time derivatives (Fig. 4a inset). For each sample, we calculate the statistic for that sample, such as the maximum growth rate. We therefore obtain a probability distribution for the statistic and report the mean and s.d. of this distribution as the best-fit value and the estimated error (0.16±0.002 h^−1^ for the maximum growth rate for the data in Fig. 4a). A similar approach applies for any statistic that can be calculated from a single growth curve (Methods).

The data for Fig. 4b are considerably noisier than the data for Fig. 4a and the spread of data is larger at short times than at long times. The magnitude of the measurement noise changes with time. We typically assume that the measurement noise can be described by a Gaussian distribution with zero mean and a constant s.d. The magnitude of the measurement noise is determined by this s.d. and the s.d. here, for the data of Fig. 4b, appears to be time dependent (it is largest at early times). To empirically estimate the relative scale of this change, we calculate the variance across replicates at each time point. We assume that the magnitude of the measurement noise is a time-independent constant multiplied by this time-dependent relative scale and we fit that constant (Methods).

### Further applications

**Figure 5:**
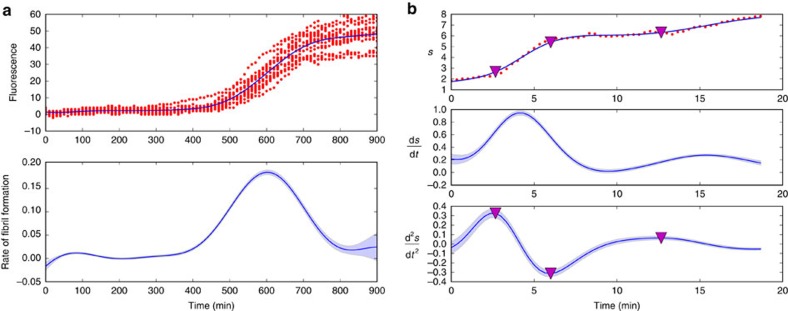
The algorithm has wide application. (**a**) Inferring the *in vitro* rate of assembly of an amyloid fibril. Fluorescence data reporting the formation of fibrils in bovine insulin (at a concentration of 0.1 mg ml^−1^) by the binding of the dye Thioflavin T are shown (red dots) with 15 replicates (each with *n*=91)^15^. The best fit (top) and the inferred rate of fibril assembly (bottom) are shown in dark blue. We empirically estimate the magnitude of the measurement noise across the replicates. Errors (in light blue) are s.d. (**b**) Inferring the speed and acceleration of separation of the spindle poles in *S. cerevisiae*. The distance, *s*, between the two spindles in a single cell is plotted in microns as a function of time (red dots with *n*=57). The best fit and the inferred speed (middle) and acceleration (bottom) are shown in dark blue. The triangles denote turning points in the acceleration and separate anaphase into stages with fast and slow elongation separated by a pause^16^. Errors are s.d.

As additional examples, we first infer the rate of assembly of an amyloid fibril as a function of time from *in vitro* data (Fig. 5a)^15^. Despite each replicate having high measurement noise compared with the microbial data, the rate of fibril assembly can be inferred accurately because of the multiple replicates. The second example is one where both the first and the second derivative are useful: estimating the speed of separation of the spindle poles during anaphase in a single cell of budding yeast (Fig. 5b). We demonstrate that we can infer both time derivatives and their errors from a single replicate. As expected, the size of the estimated error increases for the first derivative relative to the error in the regression and increases again for the second derivative. Changes in the speed of separation (extrema in the second derivative) are used to characterize anaphase^16^ into the fast, pause and slow elongation phases^17^. We chose a Gaussian process with a neural network covariance function for this data rather than the squared exponential covariance function used for the others: a difference that is important here because we only have a single replicate with few data points. The latent functions generated then tend to be flatter either side of the increase in separation, which leads to smoother inferences of the acceleration.

## Discussion

To conclude, we have introduced a non-parametric method that uses Gaussian processes to infer first and second derivatives from time-series data. In tests, our approach is at least as accurate as others (Figs 2 and 3), but has several advantages: it systematically estimates errors, both for the regression and the inferred derivatives; it allows interpolation with the corresponding error estimation (Gaussian processes were developed for interpolation^6^); and it allows sampling of the latent function underlying the data and so can be used to estimate errors in any statistic of that function by calculating the statistic for the samples.

For fitting growth curves, several alternatives exist^3^,^18^,^19^,^20^, which, although mostly focusing on parametric approaches, do allow spline fitting^3^ and polynomial regression^18^,^20^. Both approaches have been criticized, being sensitive to outliers and potentially having systematic biases^5^, and at least in the case of splines appear less robust (Fig. 3). Further, our software performs inference using all replicates, can infer second derivatives and rigorously estimates errors. Where error estimation in summary statistics has been addressed^3^, bootstrapping of the data is used. This approach is perhaps less suited for time-series data than our approach of sampling latent functions, because it leads to some randomly chosen data points being weighted more than others when generating sample fits.

Of the three we considered, we find that the squared exponential function is generally the best choice of covariance function when estimating time derivatives, because it typically results in the inference of first and second derivatives with a smoothness that is consistent with *a priori* expectations of the nature of the underlying dynamics. Although the Matern covariance is not as restrictive, because it constrains the smoothness of the latent functions less, it can lead to the inference of rough, fluctuating derivatives, in particular for the second derivative and if the magnitude of the measurement noise is high. For example, using the Matern covariance gives poor results for the data in Fig. 2a (with median error scores that are ∼60% higher than those for the squared exponential covariance), but performs slightly better (medians within 10%) for the less noisy data in Fig. 3. Finally, the neural network covariance, although perhaps the least prone to the inference of rough time derivatives, can be more sensitive to prior information: the hyperparameter controlling the flexibility of the latent function is optimized to its upper bound more often than for the other covariance functions. All three covariance functions are implemented in our code and can be tested for a new type of data.

Similar to any Bayesian method, prior information on bounds for the hyperparameters of the covariance function can affect the inference, although these bounds can typically be set so that the best-fit values are far from the bounds. In particular, how closely the latent function follows the data depends both on its flexibility and on the size of the measurement noise. An outlier can be followed if the flexibility is high or if the measurement noise is low. When there is not sufficient data, the algorithm, rightly in our opinion, requires prior information to make this choice. Alternative methods also require prior specification, such as the degree of smoothness in fitting with either splines or a local polynomial method, such as LOESS (locally weighted scatter plot smoothing). For a particular type of data, the bounds typically need to be set once allowing high-throughput analyses.

Measuring cellular growth rates is a daily task in many laboratories, but, if using a non-parameteric approach, researchers often follow the method developed in the early days of molecular biology: finding the gradient of a line fit to the portion of the growth curve that appears most straight on a semi-log plot. Our methodology takes advantages of advances in machine learning, to allow inference not only of the maximum growth rate but of the growth rate as a function of time. Time-dependent growth rates must capture more of the underlying biology, such as the time of the diauxic shift in Fig. 4a, but they have been little exploited. We believe that using more advanced inference techniques, such as the one based on Gaussian processes that we present here, in combination with developments in high-throughput technologies will transform our understanding of cellular growth and the factors that control it.

## Methods

### Using a Gaussian process to fit time-series data

In the following, we will denote a Gaussian distribution with mean *μ* and covariance matrix Σ as 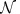
(*μ*, Σ) and use the notation of Rasmussen and Williams^6^ as much as possible.

### Prior probability

For *n* data points *y*_*i*_ at inputs *x*_*i*_ (each *x*_*i*_ is a time for a growth curve), we denote the underlying latent function as *f*(*x*). We define a covariance matrix *k*(*x*, *x*′), which has an explicit dependence on hyperparameters *θ*, and obeys


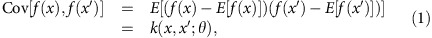


where the expectations are taken over the distribution of latent functions (samples of *f*(*x*)).

We interpret equation (1) as giving the prior probability distribution of the latent functions *f*(*X*), where were we use *X* to denote the inputs *x*_*i*_, such that


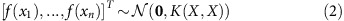


where *K*(*X*, *X*) is the *n* × *n* matrix with components *k*(*x*_*i*_, *x*_*j*_). With **f** denoting [*f*(*x*_1_), ..., *f*(*x*_*n*_)], this prior probability can be written as


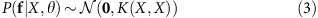


noting the dependence of *k*(*x*, *x*′; *θ*) on the hyperparameters *θ*.

### Marginal likelihood

After choosing a covariance function, to use Gaussian processes in regression, we must optimize the covariance function's hyperparameters given the observed data. We will do so by maximizing the marginal likelihood, where the marginalization is made by integrating over all possible latent functions^6^. Once the parameters of the covariance function have been determined, we can sample latent functions given the data. We consider the squared exponential, Matern (with *ν*=5/2) and neural network covariance functions.

To optimize the hyperparameters given the data, we therefore consider the likelihood *P*(**y**|*θ*, *X*), which, more correctly, is a marginal likelihood


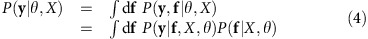


where the marginalization is over all choices of the latent function **f** evaluated at *X*.

If we assume that for all *y*_*i*_, *y*_*i*_=*f*(*x*_*i*_)+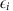 where each 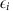 is an independent Gaussian variable with zero mean and a s.d. of *σ*_*i*_=*σ* for simplicity, then


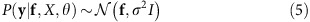


where *I* is the *n* × *n* identity matrix. Equations (3 and 5) imply that the marginal likelihood is also Gaussian:


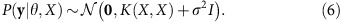


We use a maximum-likelihood method to find the hyperparameters and maximize the marginal likelihood equation (6). We have two hyperparameters for the squared exponential covariance function and the parameter, *σ*, which characterizes the measurement noise. We assume a bounded, uniform prior probability for each of these hyperparameters and use the Broyden–Fletcher–Goldfarb–Shanno algorithm^4^ to find their optimum values. Although one optimization run from random initial choices of the hyperparameters is usually sufficient, choosing the best from multiple runs can prevent the algorithm finding local maxima.

### Making predictions

Given the optimum choice of the hyperparameters, we would like to generate sample latent functions at points X*, which to include the possibility of interpolation need not be the same as *X*, by sampling from *P*(f*|*X*, **y**, *θ*, X*). Using equation (6) and that the distribution of the latent function evaluated at X* is also Gaussian, we can write the joint probability of **y** and f* as, following ref. 6,


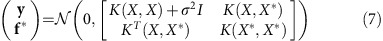


where *K*(*X*, X*) is the *n* × n* matrix with components *k*(*x*_*i*_, 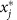).

Conditioning equation (7) on the data **y**, standard results for Gaussian distributions^6^ give that the probability distribution *P*(f*|*X*, **y**, *θ*, X*) is also Gaussian with mean


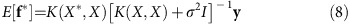


and covariance matrix


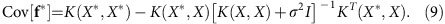


We use equations (8) and (9) to sample f*.

### Inferring the first and second time derivatives

To determine the time derivative of the data, we use that the derivative of a Gaussian process is another Gaussian process^6^. We can therefore adapt standard techniques for Gaussian process to allow time derivatives to be sampled too.

Building on the work of Boyle^7^, we let *g*(*x*) and *h*(*x*) be the first and second derivatives with respect to *x* of the latent function *f*(*x*). If *f*(*x*) is a Gaussian process then so are both *g*(*x*) and *h*(*x*). Writing *∂*_1_ and *∂*_2_ for the partial derivatives with respect to the first and second arguments of a bivariate function, we have


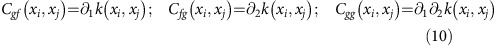


and that


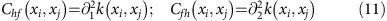


as well as


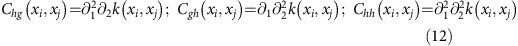


following ref. 21.

Consequently, the joint probability distribution for **y** and f*, g* and h* evaluated at points X* is again Gaussian (*cf*. equation (7)):


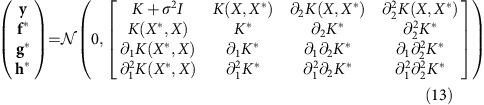


where we write *K*=*K*(*X*, *X*) and K*=*K*(X*, X*) for clarity.

The covariance function is by definition symmetric: *k*(*x*_*i*_, *x*_*j*_)=*k*(*x*_*j*_, *x*_*i*_) from equation (1). Therefore, 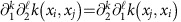 and so


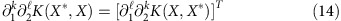


for all positive integers *k* and 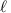. Consequently, the covariance matrix in equation (13) is also symmetric.

Conditioning on *y* now gives that the distribution *P*(f*, g*, h*|*X*, **y**, *θ*, X*) is Gaussian with mean


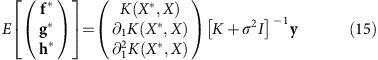


and covariance matrix


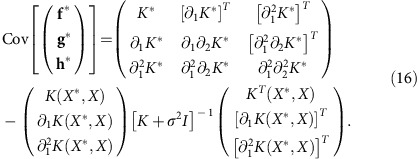


Equation (16) includes equation (9) and shows that


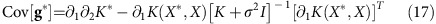


which gives the error in the estimate of the first derivative^7^. Similarly,


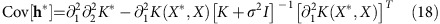


is the error in estimating the second derivative.

### Using an empirically estimated measurement noise

Although our derivation is given for a Gaussian process where the measurement errors in the data are independent and identically distributed with a Gaussian distribution of mean zero, the derivations are unchanged if the measurement noise has a different s.d. for each time point^6^.

When the magnitude of the measurement noise appears to change with time, we first empirically estimate the relative magnitude of the measurement noise by the variance across all replicates at each time point. We then smooth this estimate over time (with a Gaussian filter with a width of 10% of the total time of the experiment, but the exact choice is not important) and replace the identity matrix, *I*, in equations (6), (15) and (16) by a diagonal matrix with the relative measurement noise on the diagonal in order to make predictions.

### Estimating the growth characteristics

From the growth curve, we estimate the maximum growth rate as the maximum time derivative of the logarithm of the growth curve^2^:


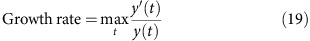


where we denote the growth curve as *y*(*t*). The doubling time is ln(2) times the inverse of the growth rate. We define the lag time as the intercept of the line parallel to the time axis that passes through the initial OD, *y*(0), and the tangent to the logarithm of the growth curve from the point on the growth curve with maximum growth rate (a standard choice^2^). If this point of maximum growth rate is at *t*=t*, then


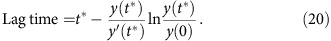


For each characteristic, we can estimate measurement error through calculating the characteristic for 100 s of sampled latent growth curves.

### Implementation and GUI

The code for our algorithm is freely available and written in Python 3 using NumPy^22^, SciPy, Matplotlib^23^, and the Pandas data analysis library (all available via the free Anaconda package) and is compatible with Microsoft's Excel. We give an example script and data set and have written a GUI that runs on Windows, OS X, and Linux.

Bounds used for the hyperparameters are given in Table 1.

### Code availability

The software and instructions for its use are at http://swainlab.bio.ed.ac.uk/software/fitderiv.

### Generating synthetic data

To generate the synthetic data shown in Fig. 3, we use a weighted sum of a Gompertz model (parameters: *A*=1.1, *μ*_m_=0.6 and *λ*=2.3; weight: 0.3) and a Richards model (parameters: *A*=1.5, *μ*_m_=0.3, *λ*=4.3 and *ν*=0.8; weight: 0.7) using the notation of ref. 2. We added log-normal measurement noise with zero mean and a s.d. of *σ*_m_=0.03 and used the SciPy implementation of a cubic spline and its time-derivative, setting the smoothing parameter to be *nσ*_m_^2^ where *n* is the number of data points.

The error score in Fig. 3 is the mean absolute deviation of the inferred growth rate from the exact growth rate ignored 5% of the data points both at the beginning and end of the time series, to avoid endpoint effects potentially dominating the error^8^.

### Experimental methods

Data for Fig. 4a was gathered using a Tecan Infinity M200 plate reader and a BY4741 strain of *Saccharomyces cerevisiae* growing in synthetic complete media supplemented with 0.4% glucose and 1% galactose at 30 °C, following an established protocol^24^. Optical density was measured at an absorbance wavelength of 595 nm every 11.4 min.

Data for Fig. 4b was gathered using a Spectrostar Omega microplate reader and a BW25113 strain of *Escherichia coli* growing in MM9 (sodium–sodium instead of sodium–potassium) media with 0.1% glucose and 1,106 mOsm sucrose at 37 °C. Optical density was measured at an absorbance wavelength of 600 nm every 7.5 min.

Data for Fig. 5a is from ref. 15.

Data for Fig. 5b was gathered using a custom spinning disk confocal microscope for 20 min in 20 s time steps with 50 ms exposure time per focal plane. Spindle pole bodies were labelled with Spc42-Cerulean. An image stack of 30 *z*-planes with 300 nm step size was gathered for each time point to allow the position of the spindle poles to be fitted to three-dimensional Gaussian distributions and tracked in time. Imaging, fitting and tracking followed an established protocol^16^.

### Data availability

Data generated in this work is available at http://dx.doi.org/10.7488/ds/1405.

## Additional information

**How to cite this article:** Swain, P. S. *et al*. Inferring time derivatives including cell growth rates using Gaussian processes. *Nat. Commun.* **7,** 13766 doi: 10.1038/ncomms13766 (2016).

**Publisher's note**: Springer Nature remains neutral with regard to jurisdictional claims in published maps and institutional affiliations.

## Figures and Tables

**Table 1:** Ranges of hyperparameters used for the examples.

**Figure**	**Covariance function**	**Hyperparameter**	**Lower bound**	**Upper bound**
1a	Squared exponential	0	10^−5^	10^5^
		1	10^−3^	10^2^
		2	10^−5^	10^2^
	Neural network	0	10^−1^	10^5^
		1	10^3^	10^3^
		2	10^−6^	10^2^
1b	Squared exponential	0	10^−5^	10^5^
		1	10^−3^	10^4^
		2	10^−5^	10^2^
	Neural network	0	10^−1^	10^5^
		1	10^1^	10^2.5^
		2	10^−6^	10^2^
2a & 2b	Squared exponential	0	10^−5^	10^5^
		1	10^−6^	10^2^
		2	10^−5^	10^2^
3a	Squared exponential	0	10^−5^	10^5^
		1	10^−6^	10^2^
		2	10^−5^	10^0^
3b	Neural network	0	10^−1^	10^5^
		1	10^−4^	10^−1^
		2	10^−6^	10^2^

For the squared exponential covariance function, the hyperparameters determine the amplitude of the variation in the latent function, its flexibility and the magnitude of the measurement noise; for the neural network covariance function, the hyperparameters determine the initial *y*-value of the latent function, its flexibility and the magnitude of the measurement noise.
